# Tracking the Effect of Phosvitin (PV) Concentration on the Skin Permeation of Somatotropin (STH) from Semi-Solid Hydrogel Formulations

**DOI:** 10.3390/polym17070980

**Published:** 2025-04-04

**Authors:** Wioletta Siemiradzka

**Affiliations:** Department of Pharmaceutical Technology, Faculty of Pharmaceutical Sciences in Sosnowiec, Medical University of Silesia, Jedności Street 10, 41-200 Sosnowiec, Poland; wsiemiradzka@sum.edu.pl

**Keywords:** somatotropin, phosvitin, skin permeation, drug carrier, hydrogels, biocompatibility, prolonged release, rheological properties, texture analysis, skin delivery

## Abstract

Recombinant human growth hormone (rhGH) is utilized in pediatric patients with short stature for a variety of indications, including those in which the primary growth defect is not related to growth hormone deficiency (GHD). However, due to the instability of the hormone in the gastrointestinal tract and its short half-life, an alternative route of administration is being sought, which may be the skin. One strategy to extend the half-life of proteins involves the use of biodegradable polymeric matrices for transdermal drug delivery systems. While hydrogels are recognized for their high stability, the transport of proteins through the skin may be hindered. To address this, the use of active carriers is being investigated to enhance the efficiency of protein permeation through the skin. In this study, an effort was made to optimize the concentration of phosphitin (PV) as a carrier for somatotropin (STH). PV is a protein that possesses a distinctive cation chelating capability and amphiphilic character. As the concentration of PV increased, the rate of its emulsifying activity increased concomitantly. Methylcellulose (MC) was used as the hydrogel matrix. The study investigated three distinct concentrations of PV to ascertain the most optimal concentration to enhance STH availability. Following the formulation of hydrogel compositions containing STH and PV, the permeation process through porcine skin was examined using Franz’s chambers. The findings revealed that the incorporation of PV significantly impacted both the penetration time of STH and the extent of STH penetration. Subsequently, an extensive evaluation of the physicochemical parameters of the formulations, encompassing pH, rheological, and textural properties, was conducted to assess their suitability for skin application. This evaluation aimed to ensure not only adequate persistence time of the formulation on the skin surface but also formulation stability and persistence of the active substance (STH).

## 1. Introduction

Since its initial introduction in 1985, recombinant human growth hormone (rhGH) has been utilized in the treatment of children with short stature for a variety of indications, including those in which the primary growth defect is not related to GH deficiency, such as Turner syndrome or Noonan syndrome [1]. The efficacy of rhGH therapy in reversing or ameliorating the numerous abnormalities associated with growth hormone deficiency (GHD) in adults has been well-documented. However, the therapeutic response to this therapy exhibits significant variability among patients and is influenced by numerous factors. The efficacy of this treatment has been enhanced, and the frequency of adverse events has been reduced, through the implementation of individualized determination of the initial and maintenance doses of rhGH. The determination of these doses is primarily based on factors such as age, the age of onset of GHD, gender, body mass index, baseline GH status, and quality of life [2]. However, the challenges associated with the short half-life, instability in the gastrointestinal tract, and the short plasma circulation time, necessitating frequent parenteral administration of rhGH, can lead to patient indiscipline and noncompliance [3,4].

In order to prolong GH exposure, the use of non-covalent albumin-binding GH compounds seems to be an interesting solution. This technology utilizes protein modification to enhance binding affinity for serum proteins. The efficacy of this technology stems from its reversible binding to serum albumin, thereby prolonging the circulation time of GH. A study was conducted to assess the efficacy, safety, and tolerability of somapacitan when administered to prepubertal children on a weekly basis at doses of 0.04, 0.08, and 0.16 mg/kg/week. In contrast, the administration of daily GH at 0.034 mg/kg/day was found to be less effective. At week 52, the efficacy of somapacitan at 0.16 mg/kg/week was found to be statistically significantly greater than that of daily GH [5,6].

Siemiradzka et al. conducted a study on the effect of albumin on STH permeation parameters through porcine skin. The incorporation of albumin as a transport protein for STH led to a substantial augmentation in skin permeation time, extending it to a minimum of 24 h. The study demonstrated that albumin enhanced the availability of STH, ranging from 80% to 93%, contingent on its concentration. Concurrently, albumin prolonged the half-life of STH, t_50%_. It was previously established that methylcellulose (MC) as a polymeric matrix effectively released STH to a satisfactory degree [7]. In the ongoing pursuit to prolong the half-life of protein drugs, the exploration of biodegradable polymer matrices has been a focal point of research for numerous years. The ideal properties of a hydrogel for use in a transdermal drug delivery system include excellent biocompatibility, biodegradability, flexibility, ease of application, a soft texture, and a high water content. The hydration effect induced on the skin by hydrogels has been demonstrated to enhance the transport of drugs through the skin [8,9,10]. The employment of polymeric materials in protein delivery systems confers numerous benefits, including simplified production methodologies, cost-effectiveness, high performance, and biocompatibility. These materials also possess low sensitizing potential and an aesthetically pleasing, contemporary delivery mechanism. A salient benefit of polymeric materials is their high stability, which enables the introduction of the active substance in a solution or suspension without the concern of sedimentation.

Protein denaturation leading to loss of stability and therapeutic activity has been documented, as well as potential toxicity and immunogenicity associated with structural changes of proteins after adsorption to solid surfaces. The adsorption of proteins to solid surfaces can result in the formation of stable complexes due to strong hydrophobic interactions or transient complexes due to hydrophobic and electrostatic interactions. Consequently, the selection of a polymer is of paramount importance in the field of protein delivery [11,12,13,14].

The only marketed long-acting Nutropin depot product that is currently available is a microsphere-based formulation composed of PLGA (polylactic-co-glycolic acid). The efficacy of a PLGA in facilitating prolonged rhGH delivery is contingent upon its capacity for protein adsorption and desorption under optimal conditions. However, studies, including those on Nutropin depot, have demonstrated inadequate rhGH loading onto PLGA microspheres [14,15,16,17].

The extant evidence suggests that among cyclodextrins (CDs), HP-β-CD (hydroxypropyl-β-cyclodextrin) has the greatest effect on GH permeation through the skin and can effectively inhibit GH degradation [18,19]. However, the utilization of β-CD is not recommended due to the formation of highly insoluble cholesterol-containing complexes and the toxicity associated with prolonged exposure to such complexes [20]. A study by Shams et al. analyzed the simultaneous administration of urea and GH using HP-β-CD as an enhancer. The most optimal results were obtained with a formulation containing 2 M urea and HP-β-CD, with an increasing trend. The study observed a maximum permeability at 120 min post-sampling, which was significantly higher than that of the control sample. Conversely, the use of 4 M urea and HP-ß-CD resulted in a significant reduction in GH release [21].

The skin serves as an alternative route for hormone delivery, thus circumventing the first-pass effects through the liver, the breakdown of the active ingredient in the gastrointestinal tract, and the interactions with food and orally administered drugs. This alternative route has been shown to result in a reduced incidence of adverse effects [22].

In recent years, significant attention has been directed toward the functional analysis of phosphopeptides as part of the daily diet. Phosphopeptides, a prominent mineral carrier, have demonstrated immunomodulatory, anti-cancer, hypoglycemic, hypolipidemic, antioxidant, and antimicrobial properties due to their biological activities associated with phosphorylated serine residues [23,24]. Phosvitin (PV) has demonstrated the capacity to chelate metal ions, including Ca^2+^, Fe^2+^, and Zn^2+^ in an acidic environment, thereby facilitating their absorption and retention in the gastrointestinal tract. The capacity of phosphopeptides to bind calcium is found to be substantially influenced by the content of phosphate groups. The majority of casein phosphopeptides are characterized by a highly polar acidic sequence, comprising three phosphoserine groups and two glutamate residues [25]. Notably, casein phosphopeptide-P5 (residues 1-25), containing four phosphate groups, has been shown to promote intestinal calcium absorption and bone development in rats. Furthermore, its amino acid composition and phosphorylated structure have been observed to significantly stimulate osteoblast proliferation and differentiation activity [26]. A study by Ren et al. reported that PV stimulated osteoblast differentiation by increasing transcription factor 2 expression, osteocalcin production, and collagen synthesis, and it also inhibited TNF-α-induced inflammation. The study indicated that the addition of PV activates both the extracellular signal-regulated kinase (ERK) and protein kinase B (AKT) signaling pathways, suggesting that PV activates osteoblast differentiation and exerts anti-inflammatory effects through the ERK and AKT pathways [27]. In addition, phosphopeptides of PV exhibited electrostatic binding to calcium via carboxyl, amino, peptide bond, and phosphate group sites, resulting in a calcium complex that demonstrated significantly higher in vitro solubility (51.18 ± 0.77%) compared to calcium lactate (41.09 ± 0.82%), CaCO3 (40.49 ± 1.35%), and d-calcium gluconate (31.18 ± 1.11%), among other calcium supplements [28].

PV peptides have been demonstrated to markedly augment calcium absorption and deposition in bone when administered in food at a dosage of 1.25–5.0 mg/g [29,30,31].

These findings suggest a promising role for PV peptides in the prevention of osteoporosis [32].

PV is a protein that contains a significant proportion of hydrophilic phosphoserine residues along with a concise hydrophobic C-terminal segment. It exhibits a unique property of containing only 10% of its amino acids in a hydrophobic configuration [33,34,35,36,37,38,39,40]. The unique cation-chelating ability and amphiphilic nature of PV are represented by its structure [41]. The emulsifying properties of PV have prompted numerous studies on its potential applications. Duan et al. [42] suggested that the rate of emulsifying activity of phosvitin increased significantly with increasing concentration.

In this study, an effort was made to utilize PV as a carrier for STH based on the author’s previous findings. The study utilized MC at a concentration of 4% as the hydrogel matrix, a choice based on the findings of Siemiradzka et al. [43]. In the preliminary study, a single concentration of PV was utilized, resulting in a modest improvement in STH penetration time. In the present study, three elevated concentrations of PV were employed to ascertain whether an escalation in concentration would enhance STH availability and facilitate a protracted penetration process.

The novelty of the study lies in its ability to design the dermal delivery of hormonal substances that require long-term use. These substances can be administered to patients in a highly convenient manner by applying the formulation to the skin, thereby circumventing the need for invasive injections. The importance of adherence to dosing regimens during therapy is underscored by the consequences of noncompliance, which can compromise both the efficacy and the efficiency of treatment. Hydrogel formulations emerge as a promising vehicle for hormones. However, the process of penetration through the skin barrier can encounter obstacles. Therefore, in this study, the author focused on using a carrier that could enhance the process of penetration through the skin. The polymer matrix utilized in this study is compatible with skin tissues, does not cause irritation, is biodegradable, does not cause allergic effects, spreads easily, and ensures the stability of the therapeutic substance.

Three distinct concentrations of PV were employed to ascertain the optimal concentration for enhancing STH availability. In accordance with the findings of Duan et al. [42], it can be hypothesized that the amphiphilic properties of the hydrogel may facilitate the permeation of hydrophilic STH through the lipophilic epidermis. Following the formulation of hydrogel compositions containing STH and PV, the permeation process through porcine skin was studied, and the physicochemical parameters of the received formulations were evaluated. These parameters encompassed pH, viscosity, rheological properties, and textural properties. The evaluation of these parameters was undertaken to ascertain the suitability of the formulations for application to the skin. This evaluation was conducted with the objective of ensuring that the formulation persisted for an adequate duration on the skin surface and that the active substance (STH) remained stable.

## 2. Materials and Methods

The MC-based vehicle (viscosity of 2% solution at 25 °C: 3755 mPa·s; Sigma Aldrich, Inc., St. Louis, MO, USA) was utilized at a concentration of 4% (4% MC). Water, in conjunction with glycerol, was subjected to heating at a temperature of 80 °C. Subsequently, MC was introduced to the mixture, and the solution was vigorously stirred with a glass dipstick until it exhibited increased viscosity. The medium was subsequently stored in a refrigerated environment maintained at 4 °C. To obtain a transparent hydrogel with MC, the substrate required a minimum of 24 h of storage before further manipulation of the base was undertaken.

Hormone—STH (Genotropin 12—somatotropin, powder for solution for injection 12 mg (36 IU), batch number HH4652, Pfizer, Europe MA EEIG, Bruxelles, Belgium) was added to the prepared base hydrogels after dissolving in a part of water and stirring until thickened. Finally, phosvitin (PV) (Sigma-Aldrich, St. Louis, MA, USA) in the form of an aqueous solution with a concentration of 1 mg/mL was introduced into the STH-loaded hydrogels in an amount corresponding to the final concentration in the formulation of 0.0005%, 0.001%, and 0.002%. The contents of the prepared hydrogels are listed in Table 1. All hydrogels were stored in a refrigerator at 4 °C.

### 2.1. Measurement of pH

The pH values of the prepared hydrogel formulations were measured using the potentiometric method described in the Polish Pharmacopoeia XII [44]. The measurement was made by directly immersing a glass electrode (In Lab Expert Pro-ISM, No. 30014096, Mettler-Toledo AG, Greifensee, Switzerland) in the hydrogel preparations.

### 2.2. Rheology Properties

A Lamy RM 200 TOUCH rotational rheometer (Lamy Rheology Instruments, Champagne au Mont d’Or, France), controlled by Rheomatic software Version: 2.1.0.4 (software from Lamy Rheology Instruments, Champagne au Mont d’Or, France), was utilized to ascertain the rheological parameters of the prepared hydrogels. The measurements were conducted using a CP 2445 plate-to-plate measuring spindle system and a CP-1 Plus thermostat. Prior to the measurements, the samples were removed from the refrigerator and placed on the bottom plate of the thermostatic system after 30 min. The determinations were made at a temperature close to that of human skin (32.0 ± 0.5 °C).

A series of experiments were conducted to assess the rheological properties of hydrogels under varying shear rates. The viscosity of the samples was measured at three distinct shear rates: 30 s^−1^, 60 s^−1^, and 100 s^−1^. To ensure comprehensive data collection, the flow curves (Flow Test) were determined within a shear rate range of D = 5–200 s^−1^, with a measurement time of 33 s. To expand the experimental scope, the shear stress versus shear rate (step-by-step method) was also measured within the shear rate range D = 5–200 s^−1^, and the measurement time was 240 s. The test was performed for a hydrogel base unloaded with protein substances and for preparations based on 4% MC, containing STH and STH with the addition of PV.

### 2.3. Texture Analysis

Texture analysis of the test formulations was carried out using a TX-700 Lamy Rheology texture analyzer (Lamy Rheology Instruments, Champagne au Mont d’Or, France) using the TPA Cycle function. The test was conducted at room temperature. A cylindrical steel sensor was used for the measurement, which was dipped twice into each sample at a speed of 1 mm/s to a depth of 10 mm. From the texture profiles, the hardness, cohesion, adhesion, elasticity, and adhesion strength of the prepared hydrogels were determined.

### 2.4. Statistical Analysis of the Results Obtained

A statistical software program (Statistica 13.0, STATSOFT; Statistica, Tulsa, OK, USA) was utilized to conduct all statistical analyses. The experimental data were expressed as the mean (M) with the standard deviation (SD). One-way analysis of variance (ANOVA) followed by the use of Tukey’s multiple comparison tests was employed to test the difference between groups; *p* < 0.05 was considered significant.

### 2.5. The Study of STH Permeation

To study the permeation of STH from the 4% MC-based hydrogel vehicle, flow-through Franz chambers made of white borosilicate glass with stirrer, type 03, 9 mm, sheathed, flat connector, type 02, 5 mL, with in/out ports in the receptor chamber, LPP Equipment Warsaw, were used. A 3-station stirrer for Franz cells with an anodized aluminum handle, LPP Equipment Warsaw, and a recirculating thermostat for the Franz diffusion cell system, LPP Equipment Warsaw, were utilized. The method of STH measurement and permeation study was described in detail in the paper by Siemiradzka during the study of STH permeation through the skin [7]. The concentration of STH in the samples was determined using a validated spectrophotometric method. From the STH spectrum obtained, recorded in the wavelength range λ = 200–400 nm, the absorption maximum of STH was determined at λ = 276 nm, at which the absorbance was measured in further determinations. Phosphate-buffered sodium chloride (PBS) at pH 7.4 was used as a reference. 0.1 mg/mL STH solution was prepared in PBS at pH 7.4, and a standard curve of STH was constructed in three replicates. STH solutions were prepared at the following concentrations: 0.1 mg/mL, 0.2 mg/mL, 0.3 mg/mL, 0.4 mg/mL, 0.5 mg/mL, 0.6 mg/mL, 0.7 mg/mL, 0.8 mg/mL, 0.9 mg/mL, and 1.0 mg/mL. Absorbance was measured, and calibration curves of absorbance versus drug concentration were plotted. The photometric accuracy of the spectrophotometer was ±0.005 A. The spectrophotometric method used was specific for the determination of the corresponding protein peptide substance. The linearity of the method was determined by 3-fold absorbance measurements of somatotropin solutions at 10 concentrations ranging from 0.01 to 1.0 mg/mL. The UV-VIS Cecil CE 3021 spectrophotometer (Cecil Instruments Limited, Cambridge, England) was utilized to conduct the measurements, and the STH content was determined through the use of a curve with the equation y = 0.8953x + 0.0032, resulting in an R^2^ value of 0.999. The amount of STH permeated was measured spectrophotometrically at a wavelength of 276 nm using a phosphate-buffered saline (PBS) solution with a pH of 7.4. The composition of the solution is as follows: sodium chloride p.a. (Chempur, Piekary Ślaskie, Poland), potassium dihydrogen phosphate (Chempur, Piekary Ślaskie, Poland), disodium hydrogen phosphate anhydrous (Chempur, Piekary Ślaskie, Poland), water for injection (Fresenius KABI, Błonie, Poland) were used as a reference. The arithmetic mean of six replicates was computed.

### 2.6. Porcine Skin Membrane for Permeation Studies

The model natural membrane, porcine skin, was obtained from a local farmer. Skin sections were trimmed to a diameter of 2.5 ± 0.5 cm^2^. Each sample was spread over the skin surface. The preparation of porcine skin is described in Siemiradzka et al. [45]. The model membrane for the permeation study was a fully grown pig ear skin obtained from a local farmer. The porcine skin was washed with a 0.9% NaCl solution to an absorbance of E < 0.02 at λ = 276 nm and dried with tissue paper. Hair was carefully trimmed with scissors to avoid damaging the stratum corneum, and the subcutaneous fat layer was removed with a scalpel. The skin samples were then protected with aluminum foil and stored at −20 °C. Skin sections were cut to a diameter of 2.5 ± 0.5 cm^2^. Each of the prepared hydrogel preparations was applied to the skin surface.

Following the application of the preparations to the membrane, the chamber was hermetically sealed to prevent evaporation of the acceptor fluid. A 5-mL volume of phosphate buffer (PBS) was introduced into the chamber via a capillary tube. Subsequently, 2-mL samples were collected at 0.5 h, 1 h, 1.5 h, 2 h, 2.5 h, 3 h, 4 h, 5 h, 6 h, and 7 h. Each collected sample was then supplemented with an additional portion of PBS. The spectrophotometer was then utilized to measure the absorbances of the collected samples at 276 nm (for STH). The experimental design involved six replicates (*n* = 6) for each sample, ensuring sufficient statistical power to draw robust conclusions. The average of six repeats was calculated.

The process of somatotropin penetration through the skin was traced by plotting the dependence of the substance concentration (C%) in the acceptor chamber against time (t). The total cumulative amount was presented as a percentage of the applied dose to the skin surface (Q) at 6 h. Based on these results, the rate constant for the somatotropin permeation process was calculated.

This is example 1 of an equation: The rate constant for the first-order reaction was calculated according to Formula (1):

k = lnC_1_ − lnC_2_/t_2_ − t_1_ (h^−1^), (1)

where:

C_1_—the total concentration of the substance in solution at time t_1_ = 0 h (%);

C_2_—total concentration of the substance in solution after time t_2_ = 3 h, 4 h, or 6 h (%).

The area under the curve (AUC) was calculated using the trapezoidal method. The AUC is a measure of the pharmaceutical availability of STH.

The correlation coefficients of the permeation kinetics of the test substance were determined using the following models: the Higuchi model, the Korsmeyer–Peppas model, and the first-order model [46].

### 2.7. Comparison of Penetration Profiles

The dissolution profiles of the different formulations of STH were compared using one-way ANOVA, using 13 Statistica (StatSoft, Cracow, Poland). *p* < 0.05 was considered statistically significant. A model-independent mathematical approach was also used to compare the dissolution profiles of the samples and the reference product using the difference factor (f1) and similarity factor (f2) [47].

## 3. Results

### 3.1. Ascertainment of pH Values

As illustrated in Table 2, the pH values of the prepared hydrogel formulations are presented.

The pH value was measured at room temperature, and the hydrogel used as a base for the active substance, containing MC at a concentration of 4% as a gel-forming substance, showed an average value of 4.31 ± 0.01. However, the loading of the hydrogel base with both the hormone substance and the substance used as a transporter for the hormone resulted in an increase in the pH value above 5, thereby ensuring the safety of formulations applied to the skin and averting any potential irritation. It was observed that the increase in pH was more pronounced in higher concentrations of protein substances STH and PV. It was observed that the addition of STH to the base prepared with 4% MC caused a significant increase in pH. However, a further increase in pH after the addition of PV was already less significant. The higher the concentration of PV, the higher the pH, reaching 5.63 ± 0.02 for STH-P1, 5.65 ± 0.02 for STH-P2, and 5.82 ± 0.01 for STH-P3. Consequently, all formulations containing STH can be applied to the skin surface without the risk of irritation.

### 3.2. Rheological Properties

The rheological properties of the prepared formulations were evaluated by measuring viscosity at three different shear rates: 30 s^−1^, 60 s^−1^, and 100 s^−1^. The measurement results are presented in Table 3.

In all prepared hydrogel formulations containing solely the gelling agent MC, as well as in formulations containing STH and STH and PV, viscosity decreased with increasing shear rate. STH and STH-P3 increased the viscosity most to values of 11,534.34 ± 271.00 (mPa·s) and 11,534.40 ± 250.52 (mPa·s) at a shear rate of D = 30 s^−1^. At shear rates of D = 60 s^−1^ and D = 100 s^−1^, the highest increase in viscosity was caused by the addition of PV at the highest concentration in the STH-P3 formulation: 7978.53 ± 64.86 (mPa·s), (D = 60 s^−1^) and 6035.30 ± 70.23 (mPa·s), (D = 100 s^−1^). The addition of PV to STH-loaded formulations resulted in a slight concentration-dependent increase in viscosity, with higher concentrations of PV leading to greater increases in viscosity. The addition of PV at a concentration of 0.001% to the 4% MC-based vehicle alone resulted in an increase in viscosity to 11,084.22 ± 39.57 (mPa·s) at D = 30 s^−1^, to 7788.78 ± 91.08 (mPa·s) at D = 60 s^−1^, and to 5986.09 ± 52.13 (mPa·s) at D = 100 s^−1^. The presence of PV at a concentration of 0.0005% resulted in an increase in viscosity to 11,251.01 ± 161.62 (mPa·s) at D = 30 s^−1^, 7925.20 ± 98.96 (mPa·s) at D = 60 s^−1^, and 5991.48 ± 57.09 (mPa·s) at D = 100 s^−1^.

**Figure 1:**
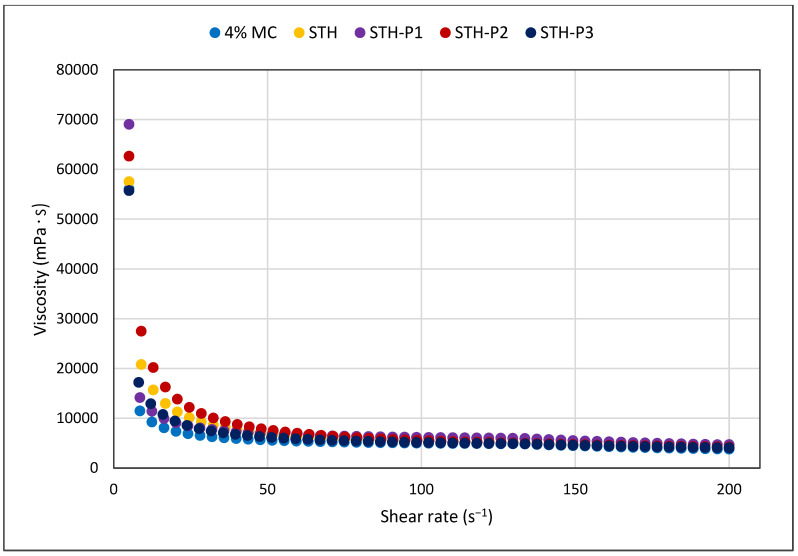
Viscosity curves for formulations unloaded hydrogel base, containing 4% MC, 4% MC and STH at concentration 0.1%, STH, and STH with the addition of PV (STH-P1 at concentration STH:PV 0.1%:0.0005%, STH-P2 at concentration STH:PV 0.1%:0.001%, and STH-P3 at concentration STH:PV 0.1%:0.002%); *n* = 6.

**Figure 2:**
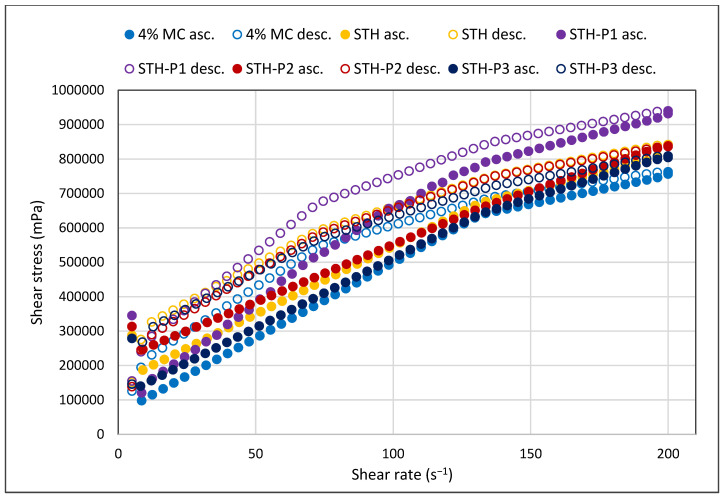
Flow curves obtained by the “FT” method of hydrogels with 4% MC, 4% MC and STH at concentrations 0.1% (STH), 4% MC and STH (0.1%) and PV (0.0005%) (STH-P1), 4% MC and STH (0.1%) and PV (0.001%) (STH-P2), and 4% MC and STH (0.1%) and PV (0.002%) (STH-P3); *n* = 6.

**Figure 3:**
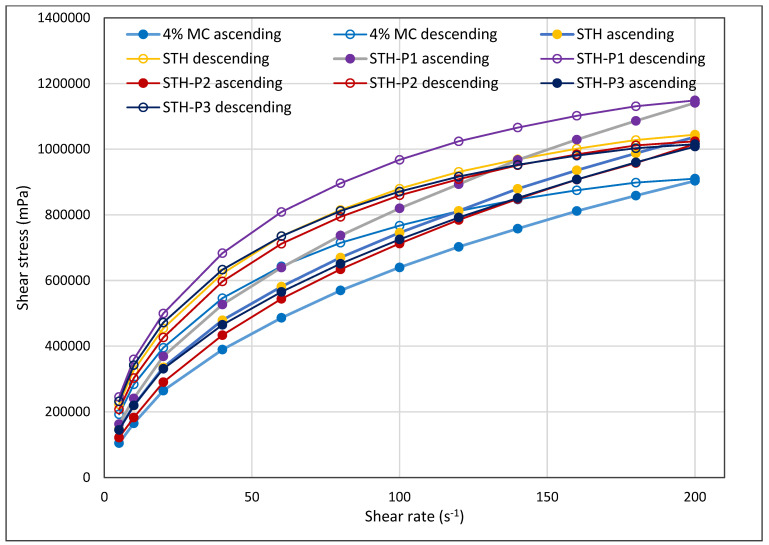
Flow curves (hysteresis loops) from the step-by-step test of hydrogels with 4% MC, 4% MC and STH at concentrations of 0.1% (STH), 4% MC and STH (0.1%) and PV (0.0005%) (STH-P1), 4% MC and STH (0.1%) and PV (0.001%) (STH-P2), and 4% MC and STH (0.1%) and PV (0.002%) (STH-P3); *n* = 6.

In all formulations containing STH, PV, and in a 4% MC-based substrate without active substances, it was demonstrated that the viscosity of hydrogels decreases with increasing shear rate (Figure 1). The systems are shear-thinning and belong to thixotropic systems, as confirmed by the shape of the flow curves in Figure 2 and by the hysteresis loop in Figure 3. The relationship between shear stress and shear rate is shown in Figure 2. The course of the flow curves of all prepared formulations indicated their non-Newtonian nature, and the relationship between shear stress and shear rate deviated from a straight line, with a shift along the ordinate in the shear rate range from 5 s^−1^ to 200 s^−1^.

Therefore, the equation for non-linear viscoelastic bodies, Casson’s Equation (2), was used to approximate the values of shear stress and shear rate:

(2) $$\sqrt{\tau }=\sqrt{\tau_{y}}+\sqrt{\eta \gamma }$$

where τ = shear stress [Pa], τ_y_ = constant interpreted as yield stress [Pa], η = experimentally determined viscosity [Pa·s], and γ = shear rate [s^−1^]. This method allows the determination of the flow limit, and its existence is indicated by the shape of the flow curve.

The experimental data were analyzed to generate flow curves, which were plotted as a function of shear stress ranging from 5.0 to 200.0 s^−1^. The resulting data were then examined to determine the shear stress dependence on shear rate, as illustrated in Figure 2. Additionally, the hysteresis loops were assessed through a step-by-step test, as depicted in Figure 3. It should be noted that the measurement duration for the flow test was 33 s, whereas the step-by-step test required a longer measurement time of 240 s.

As shown in Table 4, the area under the curve (AUC) of the melt flow curves generated by the flow curve (FT) step test (SBS) of the prepared hydrogels (MC 4%, STH, STH-P1, STH-P2, and STH-P3) is reported. The ability of the hydrogel to revert to its initial structure after the termination of the stress interactions is indicative of its rheological stabilization.

### 3.3. Texture Analysis

An analysis of the texture parameters, as shown in Table 5, revealed that the introduction of protein peptide substances resulted in a significant increase in the hardness, adhesiveness, and adhesive strength of the 4% MC-based hydrogel vehicle. Conversely, there was a decrease in cohesiveness and elasticity. The most significant effect was observed for STH. The hydrogel with STH was characterized by the highest hardness (0.435 ± 0.035 N), which was 12 times higher than that of the 4% MC base (0.035 ± 0.004 N). The inclusion of PV resulted in a decrease in hardness, adhesion, and bond strength compared to the STH-only formulation. However, these values remained significantly higher than those observed for the 4% MC base. It was observed that the introduction of PV resulted in a significant improvement in the hardness, cohesiveness, adhesion, elasticity, and tack of the formulations. This observation was further corroborated by the favorable increase in textural parameters, particularly with regard to the topical use of the formulations on the skin, as compared to the unloaded vehicle. The formulations exhibited improved spreadability on the skin surface due to increased elasticity, improved adhesion to the skin, prolonged bond strength, improved textural cohesiveness, and increased mechanical resistance.

**Figure 4:**
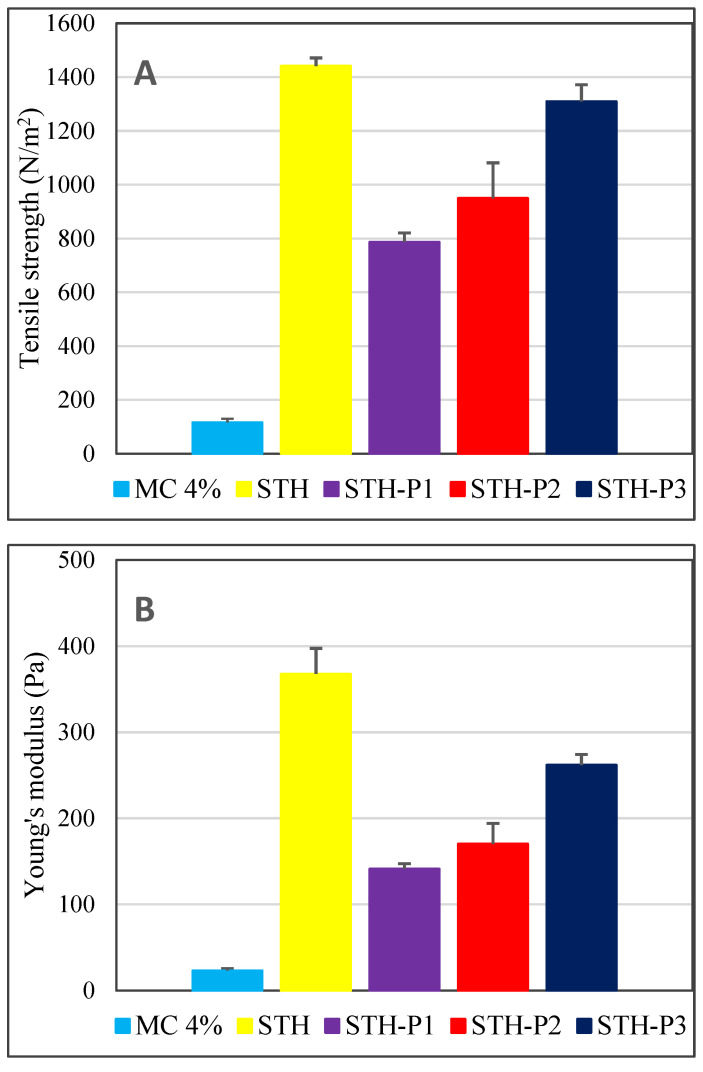
Tensile strength (**A**) and Young modulus (**B**) of prepared hydrogel formulations; *n* = 6.

Furthermore, the composition used in the formulation can be seen to influence the strength of the designed hydrogel formulation (Figure 4). The tensile strength (3) and Young’s modulus (modulus of elasticity, (Pa)) (4) were computed as follows.

(3) $$Tensile\;strength=\frac{F}{A}$$

(4) $$Young^{\prime }s\;modulus=\frac{F/A}{\Delta l/l}$$

where *F* (N) is the force applied to the hydrogel, *A* (m^2^) is the calculated cross-sectional area of the hydrogel cylinder, *Δl* (m) is the length deformation, and *l* (m) is the original sample length. Young’s modulus is a measure of the “stiffness” (mechanical response) of a material, which is the ability of the material to recover its initial shape after deformation [48].

As illustrated in Figure 4, the hydrogel containing 0.1% STH demonstrated the optimal tensile strength among the tested samples. The tensile strength of the 4% MC base vehicle shows a 12-fold enhancement, suggesting the stabilizing effect of STH on base properties. However, the combination of STH (0.1%) with PV at concentrations of 0.0005%, 0.001%, and 0.002% led to a reduction in tensile strength. Notably, PV at 0.002% exhibited the least impact on strength reduction, with only a 1.1-fold reduction observed.

Young’s modulus, denoted as the longitudinal modulus of elasticity of the system, is a quantitative measure of a material’s capacity for deformation under tensile and compressive forces. The magnitude of this modulus determines the material’s resistance to various mechanical stresses. As illustrated in Figure 4, protein substances exert a pronounced influence on the mechanical resistance of hydrogels. The formulation containing 0.1% STH exhibited the highest Young’s modulus (*YM*), reaching a value 16 times greater than that of the unloaded vehicle. In contrast, the addition of PV to STH-loaded formulations led to a concentration-dependent reduction in the *YM* value of STH-loaded hydrogels. However, this parameter remained significantly higher than that of the non-protein-loaded base containing only 4% MC.

### 3.4. The Permeation Study

**Figure 5:**
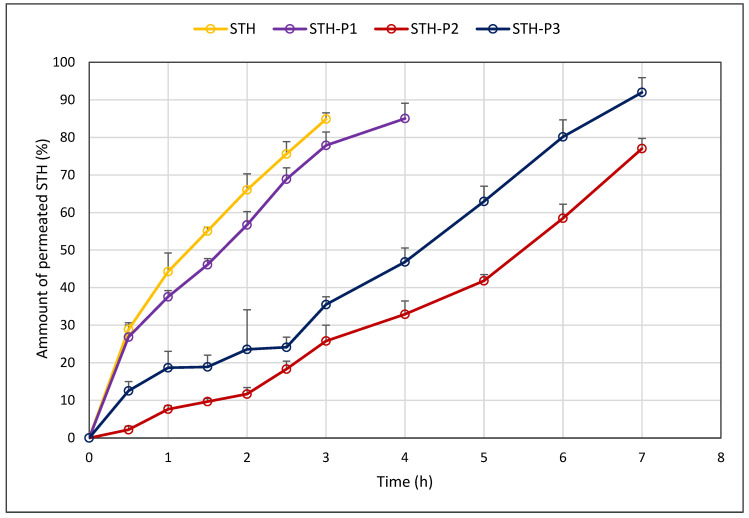
Amount of permeated STH and STH with PV Q (%) at time 6 h t [h] through porcine skin; (*n* = 6).

The process of somatotropin penetration through porcine skin was studied for 7 h. The test conditions using a natural membrane were designed to simulate in vivo conditions. The process of somatotropin permeation through porcine skin from a 4% MC base is presented in Figure 5.

The permeation process of STH from the 4% MC-based hydrogel vehicle was tracked at 7 h, and STH penetrated the skin at 3 h. After this time, no STH was observed in the collected samples. PV alters the course of STH permeation, prolonging permeation over time. The process was found to be prolonged by PV, depending on the concentration used. Specifically, it was observed to be prolonged up to 4 h (0.0005% PV) and up to at least 7 h (0.001% PV and 0.002% PV). Consequently, it can be concluded that PV may contribute to the development of STH formulations with a prolonged release process. Furthermore, the amount of STH permeating through the skin increased in a concentration-dependent manner. The extent of STH permeation through the skin increased in a concentration-dependent manner, with the highest observed concentrations of PV, specifically in the STH-P3 formulation (0.002% PV), resulting in a significant increase in the amount of STH permeated. The amount of STH penetrated, defined as the fraction of the STH dose added to the hydrogel base, increased from 84.88 ± 1.68% (STH) to 91.98 ± 3.94% (STH-P3). The addition of 0.001% PV resulted in the most substantial prolongation of STH permeation, as STH continued to be released from this formulation after a 7 h permeation process study, and the amount of STH permeated at this time was the highest recorded value, 77.04 ± 2.67%, while no STH was detected from formulations containing 0.0005% PV and 0.002% PV after 7 h in the acceptor fluid. Increased pharmaceutical availability is also indicated by the values of the STH area under the AUC permeation process curve. As illustrated in Table 6, the highest AUC value was observed for the STH formulation with the incorporation of 0.002% PV. Consequently, it can be deduced that this formulation potentially exhibits the highest availability during the study period. However, further validation is required to ascertain the prolonged release potential of the STH-P2 formulation, which would necessitate extended STH permeation study times.

The ascending and descending courses of the developed compositions remained relatively close to each other, indicating that the active ingredient, STH, did not induce any adverse changes in the hydrogel structure. Its stabilizing effect is evident, as evidenced by the hysteresis loops formed between the ascending and descending curves by flow test and step-by-step in the hydrogel with STH, which cover a smaller area than the unloaded hydrogel base (MC 4%). The magnitude of AUC for 4% MC was 35,921,571.1 (N/m^2^·s^−1^) for FT, 89,492.0 (N/m^2^·s^−1^) for SBS, and 31,498,465.2 (N/m^2^·s^−1^) for FT and 84,822.7 (N/m^2^·s^−1^) for SBS for STH, respectively. The incorporation of PV into the formulation with STH led to a modest loosening of the hydrogel structure, with the extent of loosening depending on the concentration of PV used. It was observed that an increase in the concentration of PV resulted in an enlargement of the hysteresis loop area. However, the hysteresis loop area determined by the flow test exhibited a high degree of similarity to the hysteresis loop area of the unloaded vehicle.

Protein substances such as STH incorporated into a polymeric matrix can stabilize its structure, which is very beneficial for formulations applied to the skin to ensure a sufficiently long release of the active ingredient. In the case of STH, it can be assumed that a smaller hysteresis loop area than for a substrate with 4% MC will ensure a faster return to the original structure after the removal of shear forces, for example, after spreading the formulation on the skin. PV with increasing concentration loosens the internal structure of the hydrogel with STH (STH); however, applied at the highest concentration of 0.002%, despite increasing the area of the hysteresis loop in the FT test, it did not cause a change in stability with respect to the unloaded substrate. In terms of viscosity changes due to the introduction of proteins into the hydrogel matrix, both STH and PV at a concentration of 0.002% caused an increase in viscosity, which will certainly protect the formulations from thinning when applied to the skin. At the same time, this may allow the formulation to remain on the skin for a longer period of time.

The permeation profiles of STH (0.1%) with the addition of PV at three concentrations—0.0005% (STH-P1), 0.001% (STH-P2), and 0.002% (STH-P3)—were compared with the permeation from the preparation containing STH (0.1%) without the addition of PV (STH). The amount of permeated STH Q (%) and the area under the AUC curve are shown in Table 6.

The kinetics of the STH permeation process were traced using three kinetic models: the Higuchi model, the Korsmeyer–Peppas model, and the first-order reaction model. The results are summarized in Table 7.

The regression coefficients for these models were determined, and it was ascertained that the Korsmeyer–Peppas model exhibited the highest R^2^ coefficient (R^2^ = 0.9877). The kinetics of the STH permeation process demonstrated the optimal fit to the Korsmeyer–Peppas model. The STH permeation rate and permeation constant were calculated for the first-order reaction model, as the regression coefficients calculated for the different kinetic models were relatively high for formulations containing STH as well as STH and 0.0005% PV. This is justified because PV only at higher concentrations (above 0.0005%) significantly increased the permeation time of STH. The permeation rate was highest for the STH-containing formulation (0.130 ± 0.002) and lowest for the STH-P2 formulation (0.001% PV) (0.049 ± 0.002). Changes in the STH permeation rate constant were also observed. The highest value was observed for the STH-loaded formulation (0.631 ± 0.036), while the lowest value was determined for the STH-loaded formulation (0.1%) and 0.001% PV (0.211 ± 0.017). A comparative evaluation of the profiles of the STH permeation process from the prepared hydrogel formulations was carried out. The similarity coefficients f1 and the difference coefficients f2 are outlined in Table 8, which demonstrates that the permeation process from each prepared formulation differs. The f1 and f2 values indicate that the STH permeation process differs significantly depending on the presence of PV and the PV concentration used.

## 4. Discussion

A series of hydrogel formulations were developed, with a focus on those based on 4% MC. The selection of the hydrogel base and the concentration of the polymer utilized were informed by prior studies that evaluated sodium alginate, glycerol hydrogel, and MC at varying concentrations (4% and 6%) [43].

Significant advancements have been made in the fields of cell and drug delivery, tissue engineering, and regenerative medicine, thereby establishing MC as a highly attractive and versatile biomaterial. In their work on thermo-responsive MC hydrogels, Bonetti et al. described MC as an interesting material for the preparation of thermo-responsive hydrogels, especially when body temperature is used as a trigger to modulate the thermo-responsive behavior of MC hydrogels [49].

The obtained hydrogel formulations were then subjected to a series of evaluations. These encompassed evaluations of their pH values, rheological properties, and textural characteristics.

Subsequently, the study examined the penetration of the hydrogel formulations across a natural membrane, specifically porcine skin.

In the subsequent phase of the study, the impact of incorporating PV at three distinct concentrations on the trajectory of STH permeation was examined. This investigation was undertaken with the objective of optimizing the concentration of PV. The objective of this study was to enhance the availability of STH following its application to the skin.

It is imperative that the pH of the formulation be maintained within the range of 5 to 10 to avoid the potential for skin sensitivity after use, as the recognized skin pH is approximately 5 [50]. The pH of the vehicle prepared on the basis of 4% MC was 4.31. It is imperative that the pH of the formulation be maintained. The incorporation of protein substances resulted in a favorable rise in the pH of the formulations applied to the skin surface. The pH values of the prepared hydrogels ranged from 5.56 to 5.81, indicating that all STH-containing formulations can be safely administered to the skin surface without the risk of irritancy. It was observed that the introduction of STH and STH-P3, irrespective of their concentration, resulted in a significant increase in viscosity. This phenomenon is attributed to the viscoelastic properties of the systems, which become more pronounced with an increasing shear rate. The viscoelastic nature of the semi-solid formulations received is indicative of their non-Newtonian characteristics. The distinguishing characteristic of these systems is the presence of a melt boundary. The incorporation of protein substances resulted in a modest enhancement in viscosity relative to that of the 4% MC-based vehicle alone, a phenomenon analogous to the behavior of hydrogels examined in prior studies by Siemiradzka et al. [7,43]. STH and STH-P3 exhibited the most significant increase in viscosity values. The increase in viscosity was directly proportional to the concentration of PV.

In all hydrogels prepared with STH and STH and PV, including the 4% MC-based medium without active substances, it was shown that the viscosity of the hydrogels reduces with higher shear rate. These systems exhibit shear-thinning behavior and are classified as thixotropic type, as evidenced by the shape of the flow curves.

The underlying mechanism of pseudoplasticity is believed to be the result of shear-induced enhancement, which is caused by the progressive disintegration of the liquid structure. This process is followed by a reconstruction of the configuration due to Brown’s motion, which leads to the movement of particles to the best locations from the point of view of structure entropy [51].

The capacity of a hydrogel to revert to its native structure upon the termination of stress interactions signifies its rheological stability. The incorporation of STH and PV into the polymeric matrix can stabilize its structure, a property that is particularly advantageous for formulations intended for dermal delivery. This stabilization guarantees the simplicity of application, spreadability, and release of the active substance over an extended period.

Taghizadeh, B. et al. have described the complexity of inter- and intramolecular interactions, the characteristics of the protein, and the state of the solution. These elements pose significant challenges in predicting the viscosity of a protein solution. It has been observed that an increase in protein content results in an increase in viscosity; however, this increase is not always linear. Consequently, highly concentrated hGH formulations have been found to increase the risk of forming aggregates or insoluble particles. Consequently, researchers have directed their attention toward issues related to the administration of protein injection solutions and the effect of increased viscosity on ISP (injection site pain) [52].

This study offers a novel perspective on the significance of formulation variables in parenteral growth hormone preparations and their potential impact on injection site discomfort. The subjects of this study are semi-solid preparations, distinguished by their substantial viscosity, mechanical strength, and elasticity values. Prior studies have demonstrated the stability and content of the medicinal substance over a period of four weeks for these preparations [7,43].

An analysis of the texture parameters indicated that protein peptide substances resulted in an enhancement of hardness, adhesiveness, and adhesion strength, while concomitantly reducing the cohesiveness and elasticity of the hydrogel vehicle based on 4% MC. The substantial increase in adhesiveness may indicate a protracted effect of STH, attributable to the prolonged retention of the formulations on the skin. The slight reduction in elasticity of the hydrogel compared to the unloaded base may facilitate distribution of the preparations on the dermal surface.

The study demonstrated that STH exhibited the most significant stabilizing effect on MC-base properties. Furthermore, the PV concentrations of 0.0005%, 0.001%, and 0.002% exhibited an increase in tensile strength, although to a lesser extent than that observed with STH. Young’s modulus, defined as the system’s longitudinal modulus of elasticity, is a quantitative measure of a material’s resistance to deformation, both in tension and compression. The magnitude of Young’s modulus is indicative of the material’s resilience to various mechanical stresses. The results of the study indicated that protein substances significantly enhance the stiffness of hydrogels, with STH demonstrating the most pronounced effect and PV exhibiting a slightly diminished effect. The effect of PV on the elasticity of hydrogels with STH was found to be concentration-dependent. The incorporation of PV into hydrogels with STH was found to be advantageous, as it contributed to enhancing the mechanical resistance of the hydrogels. The greatest enhancement in resilience was observed with 0.0005% PV.

The process of permeation and absorption of a bioactive substance through the skin can occur via multiple mechanisms. The active ingredient can accumulate on the skin surface, specifically in the stratum corneum, or it can be absorbed by passive diffusion into the epidermis. Furthermore, it has been observed to penetrate the dermis and the vascular-rich subcutaneous layer [22].

The penetration of the active substance is constrained by factors including the lipophilicity of the stratum corneum. Observations have revealed that this layer is predominantly penetrated by lipophilic substances (log P = 1–3), non-polar, and with a molecular weight < 500 Da [53]. Lipophilic compounds have been found to penetrate tissue structures and bind to the intercellular binder of the stratum corneum, thereby impeding their deep penetration into the skin. In contrast, hydrophilic molecules have been found to traverse moistened skin and penetrate deeply into the dermis [54]. The rate of penetration was observed to increase with increasing concentration of the active ingredient. Skin metabolism has been shown to exert a minor influence on the penetration of hormones. The epidermis contains cytochrome P450 enzymes, epoxide hydrolase, N-acetyltransferase, glucuronyltransferase, and sulfatase. The dermal activity of cytochrome P450 (CYP) enzymes constitutes 1–5% of the hepatic activity, while that of transferases is approximately 10% [55]. To avert fluctuations in the concentration of the active ingredient in the skin, it is imperative to ensure that the gel remains on the skin for an adequate duration [22].

Human growth hormone (HGH or somatotropin) is a 22-kDa single-chain peptide containing 191 amino acids, two disulfide bonds (Cys53-Cys165, Cys182-Cys189), and four alpha helices. The production of human growth hormone (HGH) is primarily attributed to the somatotropic cells residing within the anterior lobe of the pituitary gland. The secretion of this hormone occurs in a pulsatile manner, with a frequency ranging from four to eight batches of 0.5 to 0.8 milligrams per day. This characteristic is instrumental in eliciting its metabolic and anabolic effects. It is noteworthy that the isoelectric point of HGH approaches a value of 4.9 [52,56,57,58].

Following a 7 h permeation test, it was determined that STH penetrated the skin within 3 h. The PV intervention led to alterations in the course of the STH permeation process, resulting in modifications to the rate of permeation over time. The extent of this prolongation varied depending on the concentration of PV utilized, ranging from 3 h (0.0005% PV) to 7 h (0.002% PV). Furthermore, the permeation test demonstrated that STH was not fully released after the addition of 0.001% PV, whereas no STH was detected from the 0.0005% PV and 0.002% PV formulations after 7 h in the acceptor fluid. The findings indicate a direct correlation between higher PV concentrations and increased STH permeation through the skin. A substantial increase in STH levels was observed after 7 h at the highest PV concentration of 0.002% (91.98 ± 3.94%). The increase in drug availability is further substantiated by the STH AUC values, which were most elevated for the 0.002% PV STH formulation.

The regression coefficients were determined using three kinetic models: the Higuchi model, the Korsmeyer–Peppas model, and the first-order reaction model. The Korsmeyer–Peppas model exhibited the highest regression coefficients (R^2^ = 0.9877). Consistent results for STH permeation kinetics were reported in the study by Siemiradzka et al. [43].

The rate of STH permeation and the permeation constant for the first-order reaction model were calculated, as the regression coefficients calculated for different kinetic models were relatively high for formulations containing STH and STH and 0.0005% PV. This outcome is rational, given that PV only at higher concentrations (above 0.0005%) significantly augmented STH permeation time. The highest STH permeation rate was observed for the STH-containing formulation (0.130 ± 0.002), while the lowest was observed for the STH-P2 formulation (0.001% PV—0.049 ± 0.002). A parallel tendency was discerned in the alterations of the permeation rate constant of STH. The highest value of the permeation rate constant was observed for the STH-loaded formulation (0.631 ± 0.036), while the lowest value was observed for the formulation loaded with STH and 0.001% PV (0.211 ± 0.017).

A clear distinction was observed in the permeation profiles of the various formulations. The f1 and f2 values indicate that the STH permeation process varies significantly based on the presence of PV and the PV concentration employed.

The discussion of the kinetics of STH permeation through the skin is a difficult issue. The establishment of a reproducible bioequivalent in vitro release method is challenging due to numerous factors, including peptide degradation, variable burst release, the complex mechanism of multiphasic drug release with different release rates for each phase, and the sensitivity of in vitro and in vivo release profiles to the manufacturing process and the route of application, particularly subcutaneous [59]. These variations in PK properties can have a significant impact on pharmacodynamics and must be deliberately designed for each specific therapy [60].

The process of STH release and permeation through the natural membrane (porcine skin) is influenced by numerous factors, making it challenging to clearly assign a single kinetic model for STH preparations available on the market. Moreover, addressing this issue is particularly arduous due to its insufficient exploration by researchers. The extant literature on the subject is limited to a single reference, which appears in the author’s earlier work on the STH permeation process. In this study, the author compared the effect of PV with that of albumin on the process. The study revealed that both proteins contributed to prolonging the STH release time, highlighting the influence of polymer concentration, other excipients such as glycerol, and protein concentration on the process. The obtained preparations are a relatively stable form of the drug, as evidenced by stability studies conducted in previous works [7,43], which found, among other things, no reduction in the content of the active substance (STH). The technology for obtaining the preparations prepared in the work of semi-solid hydrogels is relatively simple and inexpensive; therefore, it is worthwhile to make further attempts to develop effective preparations for application to the skin, providing therapeutic concentrations of proteins at the desired level. This would represent a substantial advancement in a period in which only intravenous or subcutaneous formulations are commercially available.

## 5. Conclusions

An investigation into the process of STH permeation through a natural membrane (porcine skin) revealed that methylcellulose, at an applied concentration of 4%, facilitated a satisfactory degree of STH permeation. The release of STH from the hydrogel base and its subsequent penetration of the skin occurred within a duration of three hours. The addition of PV resulted in a significant increase in the penetration time of STH, allowing the active substance to remain on the skin long enough to ensure the gradual achievement of therapeutic concentrations. The permeation time of STH in the presence of PV is contingent upon the concentration of PV utilized. The formulation with PV at a concentration of 0.001% yielded the greatest amount of STH release. The study was conducted for a duration of 7 h, and no decline in the amount of STH was observed after 7 h. The highest STH penetration was observed in the formulation containing PV at a concentration of 0.002%, and the maximum amount of STH penetrated after 7 h. MC, when utilized as a hydrogel vehicle, exhibited favorable physicochemical properties for STH, providing the appropriate pH and adequate rheological and textural parameters, thereby enabling the development of formulations with sufficient shelf life. The addition of protein substances did not compromise these properties, and STH appeared to exert a favorable effect on the quality, rheological stability, and mechanical resistance of semi-solid formulations. In light of therapeutic and application considerations, it can be concluded that the formulations will fulfill their function as systems for prolonged and effective delivery of the peptide substance STH after application of the formulations to the skin.

## Figures and Tables

**Table 1:** Summary of ingredients of prepared hydrogel preparations.

Hydrogel Ingredients (g)	4% MC	STH	STH-P1	STH-P2	STH-P3
Methylcellulose	4.0	4.0	4.0	4.0	4.0
Glycerol 85%	10.0	10.0	10.0	10.0	10.0
STH	-	0.1	0.1	0.1	0.1
Phosvitin	-	-	5 × 10^−4^	0.001	0.002
Water	up to 100.0	up to 100.0	up to 100.0	up to 100.0	up to 100.0

4% MC—hydrogel with 4% methylcellulose, unloaded base; STH—hydrogel based on 4% MC with 0.1% STH; STH-P1—hydrogel based on 4% MC with 0.1% STH and 0.0005% PV; STH-P2—hydrogel based on 4% MC with 0.1% STH and 0.001% PV; STH-P3—hydrogel based on 4% MC with 0.1% STH and 0.002% PV.

**Table 2:** The mean pH values with SD of prepared hydrogels (*n* = 3).

Formulation Code	1	2	3	Average ± SD
4% MC	4.31	4.30	4.31	4.31 ± 0.01
STH	5.56	5.56	5.56	5.56 ± 0.00 ^1^
STH-P1	5.64	5.61	5.63	5.63 ± 0.02 ^1^
STH-P2	5.67	5.64	5.63	5.65 ± 0.02 ^1^
STH-P3	5.81	5.82	5.82	5.82 ± 0.01 ^1^

^1^—A statistically significant difference was observed in relation to the 4% MC-based hydrogel, with a *p* < 0.05.

**Table 3:** The viscosity (mPa·s) of the prepared material was measured at a temperature of 32 °C. The measurement was conducted at three distinct shear rates: 30 s^−1^, 60 s^−1^, and 100 s^−1^.

Formulation Code	Viscosity (mPa·s)		
	Shear Rate = 30 s^−1^	Shear Rate = 60 s^−1^	Shear Rate = 100 s^−1^
4% MC	9986.23 ± 261.92	6940.04 ± 153.07	5334.10 ± 55.45
STH	11,534.34 ± 271.00 ^1^	7787.05 ± 138.00 ^1^	5955.59 ± 135.60 ^1^
STH-P1	11,084.22 ± 39.57 ^1^	7788.78 ± 91.08 ^1^	5986.09 ± 52.13 ^1^
STH-P2	11,251.01 ± 161.62 ^1^	7925.20 ± 98.96 ^1^	5991.48 ± 57.09 ^1^
STH-P3	11,534.40 ± 250.52 ^1^	7978.53 ± 64.86 ^1^	6035.30 ± 70.23 ^1^

^1^—A statistically significant difference was observed in relation to the 4% MC-based hydrogel, with a *p* < 0.05.

**Table 4:** AUC of the flow courses achieved in the FT test and SBS of the prepared hydrogels MC 4%, STH, STH-P1, STH-P2, and STH-P3.

Formulation Code	AUC of the Hysteresis Loop $(\frac{N}{m^{2}}\cdot s^{-1})$	
	FT Test	SBS Test
4% MC	35,921,571.1	89,492.0
STH	31,498,465.2	84,822.7
STH-P1	33,748,968.0	93,305.0
STH-P2	33,920,707.0	95,273.1
STH-P3	35,929,417.5	95,809.1

**Table 5:** Texture analysis parameters determined by TPA test of prepared formulations (*n* = 6).

Formulation	Hardness (N)	Cohesiveness	Adhesiveness (mJ)	Elasticity	Adhesion Force F_min_ (N)
MC 4%	0.035 ± 0.004	3.046 ± 0.333	0.371 ± 0.049	1.001 ± 0.001	−0.034 ± 0.003
STH	0.435 ± 0.035 ^1^	0.401 ± 0.073 ^1^	2.133 ± 0.058 ^1^	0.900 ± 0.173	−0.344 ± 0.028 ^1^
STH-P1	0.237 ± 0.010 ^1^	0.596 ± 0.039 ^1^	1.667 ± 0.058 ^1^	0.723 ± 0.016 ^1^	−0.293 ± 0.038 ^1^
STH-P2	0.286 ± 0.040 ^1^	0.627 ± 0.024 ^1^	1.733 ± 0.058 ^1^	0.816 ± 0.060 ^1^	−0.321 ± 0.048 ^1^
STH-P3	0.395 ± 0.019 ^1^	0.524 ± 0.024 ^1^	2.333 ± 0.115 ^1^	0.900 ± 0.173	−0.387 ± 0.017 ^1^

^1^ statistically significant difference with respect to the hardness of the hydrogel with methylcellulose 4% (*p* < 0.05).

**Table 6:** The mean amounts Q (%) of permeated STH (0.1%) and STH (0.1%) with PV at three concentrations: 0.0005% (STH-P1), 0.001% (STH-P2), and 0.002% (STH-P3) at time 6 h through porcine skin (*n* = 6).

Time [h]	Amount of STH Penetrated (%) Through Porcine Skin			
	STH	STH-P1	STH-P2	STH-P3
0.5	28.86 ± 1.75	26.85 ± 3.26	2.18 ± 0.98	12.53 ± 2.46
1	44.28 ± 4.98	37.58 ± 1.68	7.66 ± 0.97	18.68 ± 4.35
1.5	55.10 ± 1.01	46.12 ± 1.61	9.67 ± 0.83	18.92 ± 3.14
2	66.03 ± 4.27	56.71 ± 3.53	11.68 ± 1.75	23.58 ± 2.70
2.5	75.60 ± 3.27	68.90 ± 3.00	18.34 ± 2.10	24.13 ± 2.07
3	84.88 ± 1.68	77.88 ± 3.56	25.81 ± 4.20	35.50 ± 5.57
4	-	85.04 ± 4.10	32.92 ± 3.55	46.85 ± 3.74
5	-	-	41.83 ± 1.68	62.96 ± 4.05
6	-	-	58.48 ± 3.73	80.15 ± 4.51
7	-	-	77.04 ± 2.67	91.98 ± 3.94 ^1^
AUC_(0-nh)_	156.17 ± 4.99	217.51 ± 2.52 ^1^	215.88 ± 2.48 ^1^	311.51 ± 17.98 ^1^

^1^ A statistically significant difference in terms of the amount of STH permeated (*p* < 0.05).

**Table 7:** STH permeation R^2^ regression coefficients, permeation rates, and rate permeation constants for first-order kinetics for the made formulations: STH, STH-P1, STH-P2, and STH-P3 at time 6 h through porcine skin (*n* = 6).

Formulation Code	Higuchi’s Model	Korsmeyer–Peppas Model	“1” Order Model	Permeation Rate [mg/cm^2^/h]	First-Order Rate Permeation
	R^2^				
STH	0.9984	0.9996	0.9777	0.130 ± 0.002	0.631 ± 0.036
STH-P1	0.9837	0.9883	0.9832	0.096 ± 0.005 ^1^	0.455 ± 0.068 ^1^
STH-P2	0.9067	0.9860	0.8781	0.049 ± 0.002 ^1^	0.211 ± 0.017 ^1^
STH-P3	0.9044	0.9770	0.8606	0.059 ± 0.002 ^1,2^	0.346 ± 0.021 ^1,2^

^1^ Statistically significant difference in relation to the STH permeation rate (*p* < 0.05). ^2^ Statistically significant differences in relation to the permeation rate of STH-P1 and STH-P2 (*p* < 0.05).

**Table 8:** Pairwise comparison of the similarity factors f1 and f2 of the STH release profiles.

Preparation	f1	f2	Dissolution Profile
STH vs. STH-P1	35.42	171.952	Dissimilar
STH vs. STH-P2	171.46	185.39	Dissimilar
STH vs. STH-P3	141.89	186.99	Dissimilar
STH-P1 vs. STH-P2	117.06	184.49	Dissimilar
STH-P1 vs. STH-P3	113.76	185.59	Dissimilar
STH-P2 vs. STH-P3	45.40	164.62	Dissimilar

STH—MC (4%) with STH (0.1%); STH-P1—MC (4%) with STH (0.1%) and PV (0.0005%); STH-P2—MC (4%) with STH (0.1%) and PV (0.001%); STH-P3—MC (4%) with STH (0.1%) and PV (0.002%).

## Data Availability

The data presented in this study are available upon request from the author (corresponding author).
